# Supplementary material for: Visualizing chaperone-mediated multistep assembly of the human 20S proteasome
Source: Nat Struct Mol Biol. 2024 Apr 10;31(8):1176–88. doi: 10.1038/s41594-024-01268-9 (PMC11327110; doi:10.1038/s41594-024-01268-9)
Supplement: Supplementary file 1 — Reporting Summary [file 41594_2024_1268_MOESM1_ESM.pdf]

nature portfolio

Corresponding author(s): Frank Adolf
John Hanna
Brenda A. Schulman

Last updated by author(s): Nov 3, 2023

Reporting Summary

Nature Portfolio wishes to improve the reproducibility of the work that we publish. This form provides structure for consistency and transparency in reporting. For further information on Nature Portfolio policies, see our Editorial Policies and the Editorial Policy Checklist.

Statistics

For all statistical analyses, confirm that the following items are present in the figure legend, table legend, main text, or Methods section.

n/a	Confirmed	
☐	☒	The exact sample size (*n*) for each experimental group/condition, given as a discrete number and unit of measurement
☐	☒	A statement on whether measurements were taken from distinct samples or whether the same sample was measured repeatedly
☒	☐	The statistical test(s) used AND whether they are one- or two-sided<br>*Only common tests should be described solely by name; describe more complex techniques in the Methods section.*
☒	☐	A description of all covariates tested
☒	☐	A description of any assumptions or corrections, such as tests of normality and adjustment for multiple comparisons
☒	☐	A full description of the statistical parameters including central tendency (e.g. means) or other basic estimates (e.g. regression coefficient) AND variation (e.g. standard deviation) or associated estimates of uncertainty (e.g. confidence intervals)
☒	☐	For null hypothesis testing, the test statistic (e.g. *F*, *t*, *r*) with confidence intervals, effect sizes, degrees of freedom and *P* value noted<br>*Give P values as exact values whenever suitable.*
☒	☐	For Bayesian analysis, information on the choice of priors and Markov chain Monte Carlo settings
☒	☐	For hierarchical and complex designs, identification of the appropriate level for tests and full reporting of outcomes
☒	☐	Estimates of effect sizes (e.g. Cohen's *d*, Pearson's *r*), indicating how they were calculated

Our web collection on statistics for biologists contains articles on many of the points above.

Software and code

Policy information about availability of computer code

Data collection	Cryo-EM: SerialEM v4.1; Gel imaging: Amersham Imager 600
Data analysis	Cryo-EM: CryoSparc v4.2.0; Structure Analysis and Visualization: Chimera v1.15, ChimeraX v1.4, DeepEMhancer (https://github.com/rsanchezgarc/deepEMhancer); Model Building: COOT v0.9.6, Phenix.refine v1.19.2-4158, AlphaFold2, ISOLDE 1.4, Clustal Omega

For manuscripts utilizing custom algorithms or software that are central to the research but not yet described in published literature, software must be made available to editors and reviewers. We strongly encourage code deposition in a community repository (e.g. GitHub). See the Nature Portfolio guidelines for submitting code & software for further information.

Data

Policy information about availability of data

All manuscripts must include a data availability statement. This statement should provide the following information, where applicable:

- Accession codes, unique identifiers, or web links for publicly available datasets
- A description of any restrictions on data availability
- For clinical datasets or third party data, please ensure that the statement adheres to our policy

The atomic coordinates and electron microscopy maps have been deposited in the PDB with accession code 8QYJ, 8QYL, 8QYM, 8QZ9, 8QYN, 8QYS and 8QYO and in the Electron Microscopy Data Bank with codes EMD-18755 for 20S assembly intermediate map 1, EMD-18757 for 20S assembly intermediate map 2, EMD-18758 for 20S assembly intermediate map 3, EMD-18773 for 20S assembly intermediate map 4, EMD-18759 for 20S assembly intermediate map 5, EMD-18761 for

preholo-20S core particle, EMD-18762 for premature-20S core particle, EMD-18760 for mature 20S core particle, EMD-19342 for beta 7 tagged half 20S core particle and EMD-19343 for beta 7 tagged mature 20S core particle. All other reagents and data (for example, raw gels of experiments and raw movie electron microscopy data) are available from the corresponding authors upon request.

Research involving human participants, their data, or biological material

Policy information about studies with human participants or human data. See also policy information about sex, gender (identity/presentation), and sexual orientation and race, ethnicity and racism.

Reporting on sex and gender	No research involving human participants has been performed
Reporting on race, ethnicity, or other socially relevant groupings	No research involving human participants has been performed
Population characteristics	No research involving human participants has been performed
Recruitment	No research involving human participants has been performed
Ethics oversight	No research involving human participants has been performed

Note that full information on the approval of the study protocol must also be provided in the manuscript.

Field-specific reporting

Please select the one below that is the best fit for your research. If you are not sure, read the appropriate sections before making your selection.

☒ Life sciences ☐ Behavioural & social sciences ☐ Ecological, evolutionary & environmental sciences

For a reference copy of the document with all sections, see nature.com/documents/nr-reporting-summary-flat.pdf

Life sciences study design

All studies must disclose on these points even when the disclosure is negative.

Sample size	Sample size calculations were not preformed.
Data exclusions	No data were excluded.
Replication	Three indpendent replicates were carried out for each purification. All attempts of replication were successful.
Randomization	No grouped samples.
Blinding	No grouped samples.

Reporting for specific materials, systems and methods

We require information from authors about some types of materials, experimental systems and methods used in many studies. Here, indicate whether each material, system or method listed is relevant to your study. If you are not sure if a list item applies to your research, read the appropriate section before selecting a response.

Materials & experimental systems

n/a	Involved in the study	
☒	☐	Antibodies
☐	☒	Eukaryotic cell lines
☒	☐	Palaeontology and archaeology
☒	☐	Animals and other organisms
☒	☐	Clinical data
☒	☐	Dual use research of concern
☒	☐	Plants

Methods

n/a	Involved in the study	
☒	☐	ChIP-seq
☒	☐	Flow cytometry
☒	☐	MRI-based neuroimaging

Eukaryotic cell lines

Policy information about cell lines and Sex and Gender in Research

Cell line source(s)	High five cell (BTI-TN-5B1-4) were obtained from ThermoFisher Scientific (catalogue number:B85502). Gibco Sf9 cells were obtained ThermoFisher Scientific (catalogue number:11496016).
Authentication	Cell lines were not authenticated.
Mycoplasma contamination	Cell lines were periodically tested for mycoplasm contamination with no contamination detected.
Commonly misidentified lines (See ICLAC register)	No commonly misidentified cell lines were used in this study.
