# Supplementary figures and images for: Visualizing chaperone-mediated multistep assembly of the human 20S proteasome
Source: Nat Struct Mol Biol. 2024 Apr 10;31(8):1176–88. doi: 10.1038/s41594-024-01268-9 (PMC11327110; doi:10.1038/s41594-024-01268-9)

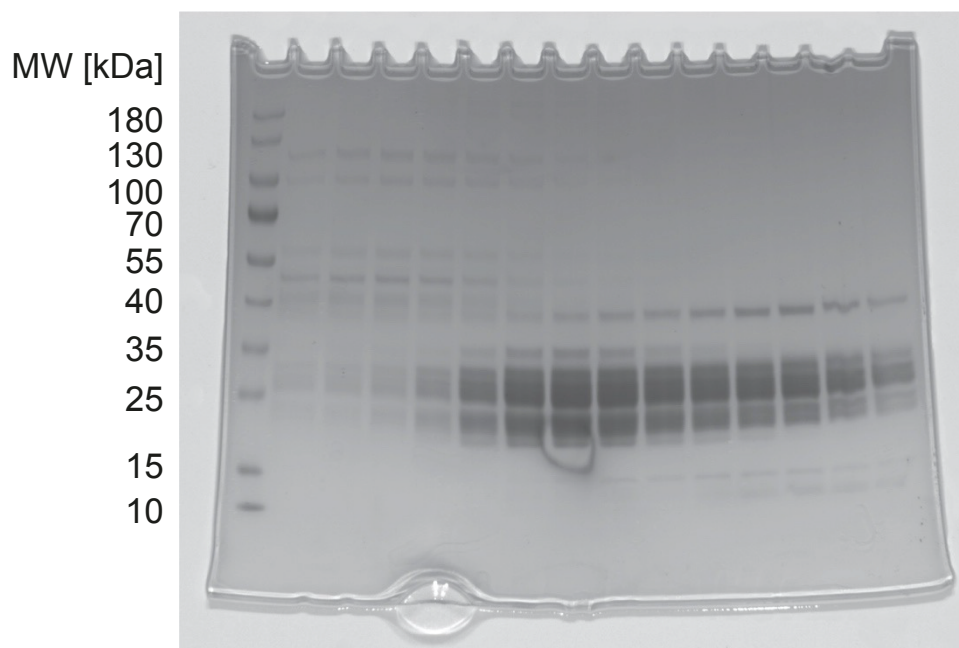

Figure 1a

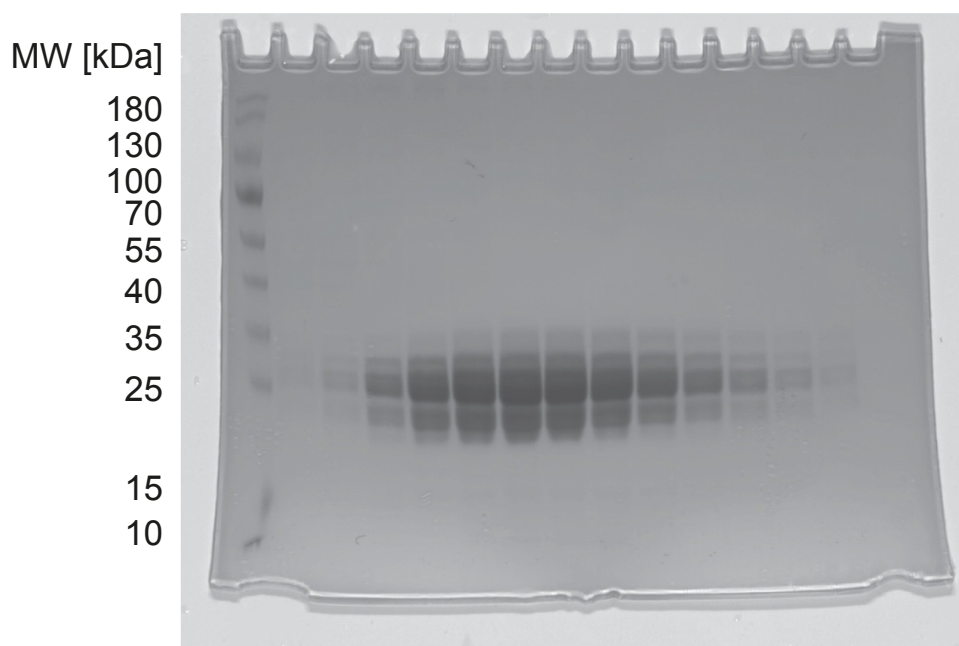

Figure 1b

Supplement: Supplementary file 14 — Unprocessed gels. [file 41594_2024_1268_MOESM14_ESM.pdf]
